# mHealth Technology and Nurse Health Coaching to Improve Health in Diabetes: Protocol for a Randomized Controlled Trial

**DOI:** 10.2196/resprot.9168

**Published:** 2018-02-15

**Authors:** Sheridan Miyamoto, Madan Dharmar, Sarina Fazio, Yajarayma Tang-Feldman, Heather M Young

**Affiliations:** ^1^ College of Nursing The Pennsylvania State University University Park, PA United States; ^2^ Department of Pediatrics University of California Davis Sacramento, CA United States; ^3^ Betty Irene Moore School of Nursing University of California Davis Sacramento, CA United States

**Keywords:** randomized controlled trial, study protocol, mobile health, health coaching, motivational interviewing, type 2 diabetes mellitus, patient generated health data, electronic health record, patient engagement, person-centered outcomes research

## Abstract

**Background:**

Chronic diseases, including diabetes mellitus, are the leading cause of mortality and disability in the United States. Current solutions focus primarily on diagnosis and pharmacological treatment, yet there is increasing evidence that patient-centered models of care are more successful in improving and addressing chronic disease outcomes.

**Objective:**

The objective of this clinical trial is to evaluate the impact of a mobile health (mHealth) enabled nurse health coaching intervention on self-efficacy among adults with type-2 diabetes mellitus.

**Methods:**

A randomized controlled trial was conducted at an academic health system in Northern California. A total of 300 participants with type-2 diabetes were scheduled to be enrolled through three primary care clinics. Participants were randomized to either usual care or intervention. All participants received training on use of the health system patient portal. Participants in the intervention arm received six scheduled health-coaching telephone calls with a registered nurse and were provided with an activity tracker and mobile application that integrated data into the electronic health record (EHR) to track their daily activity and health behavior decisions. All participants completed a baseline survey and follow-up surveys at 3 and 9 months. Primary and secondary outcomes include diabetes self-efficacy, hemoglobin A_1c_ (HbA_1c_), and quality of life measures.

**Results:**

Data collection for this trial, funded by the Patient-Centered Outcomes Research Institute, will be completed by December 2017. Results from the trial will be available mid-2018.

**Conclusions:**

This protocol details a patient-centered intervention using nurse health coaching, mHealth technologies, and integration of patient-generated data into the EHR. The aim of the intervention is to enhance self-efficacy and health outcomes by providing participants with a mechanism to track daily activity by offering coaching support to set reasonable and attainable health goals, and by creating a complete feedback loop by bringing patient-generated data into the EHR.

**Trial Registration:**

ClinicalTrials.gov NCT02672176; https://clinicaltrials.gov/ct2/show/NCT02672176 (Archived by WebCite at http://www.webcitation.org/6xEQXe1M5)

## Introduction

Diabetes mellitus is a global epidemic highly amenable to health promotion interventions. Over 29 million Americans are currently living with diabetes. Since 2000, approximately 1 million new cases are diagnosed each year, with type-2 diabetes accounting for 80-95% of cases [1,2]. Uncontrolled diabetes can lead to major vascular complications including heart disease and stroke, hypertension, blindness, lower limb amputations, peripheral neuropathy, lipid abnormalities, and kidney disease [3-6] and can have a profound impact on quality of life and functional ability. Promotion of self-management strategies such as healthy eating, being physically active, monitoring blood glucose, medication adherence, stress management, and healthy coping are essential for preventing adverse consequences of diabetes [7].

Health interventions that involve active patient engagement have sustained and improved clinical and psychosocial outcomes over didactic interventions with limited patient input [8-12]. Traditional offerings for diabetes management typically emphasize education and do not address patient-centered goals and personal motivations [9]. Diabetes management in the electronic health record (EHR) is episodic and provider-centered, focused on diagnostic, clinical, and pharmaceutical records, with no process to capture and review patient-centered goals or patient-generated health data (PGHD). A vital shift is needed to actively involve patients in developing their care plan and to effectively highlight patient-centered priority areas in the EHR with the health care team.

In the Patient and Provider Engagement and Empowerment through Technology (P^2^E^2^T^2^) to Improve Health in Diabetes study, we sought to design and test an intervention to enhance self-efficacy of diabetes self-management for persons living with type-2 diabetes mellitus. In collaboration with persons living with diabetes, healthcare providers, and technology stakeholders, we created a patient-centered intervention with the following components: 1) A wearable mobile activity tracker and nutrition apps; 2) Nurse health coaching sessions; and 3) Integration of patient-generated daily activity data into the EHR. This protocol describes the randomized controlled trial designed to test the impact of the P^2^E^2^T^2^ Program to Improve Health in Diabetes intervention on self-efficacy compared to those who receive usual care.

### Intervention Design: Pilot Data and Key Stakeholder Partnership

Several previous studies established the basis for the current protocol, exploring design features and testing feasibility and efficacy of elements of the intervention.

#### Nurse Health Coaching and Sustained Self-Efficacy

Our team has studied the effect of nurse health coaching in a population of people living with type-2 diabetes. In a previous randomized experimental study comparing nurse health coaching to usual care, we offered the intervention group 6 nurse health coaching sessions, occurring approximately every 2 weeks, over a 3-month period. The nurses based coaching sessions on the principles of motivational interviewing, a patient-centered counseling technique used in many disciplines to support behavior change and enhance self-efficacy [13,14]. A total of 121 participants were enrolled in the study. Of those, 101 completed all 3 measurements of self-efficacy at baseline, 3 months, and 9 months and were included in the analysis. Results demonstrated sustained effects of the intervention with a significant difference in self-efficacy scores at 9 months among those who received nurse health coaching relative to the control group [15]. A limitation of this study was the lack of objective patient-generated data about goal attainment, including physical activity and nutritional outcomes.

#### Tracking Health Data is not Enough

**Figure 1:**
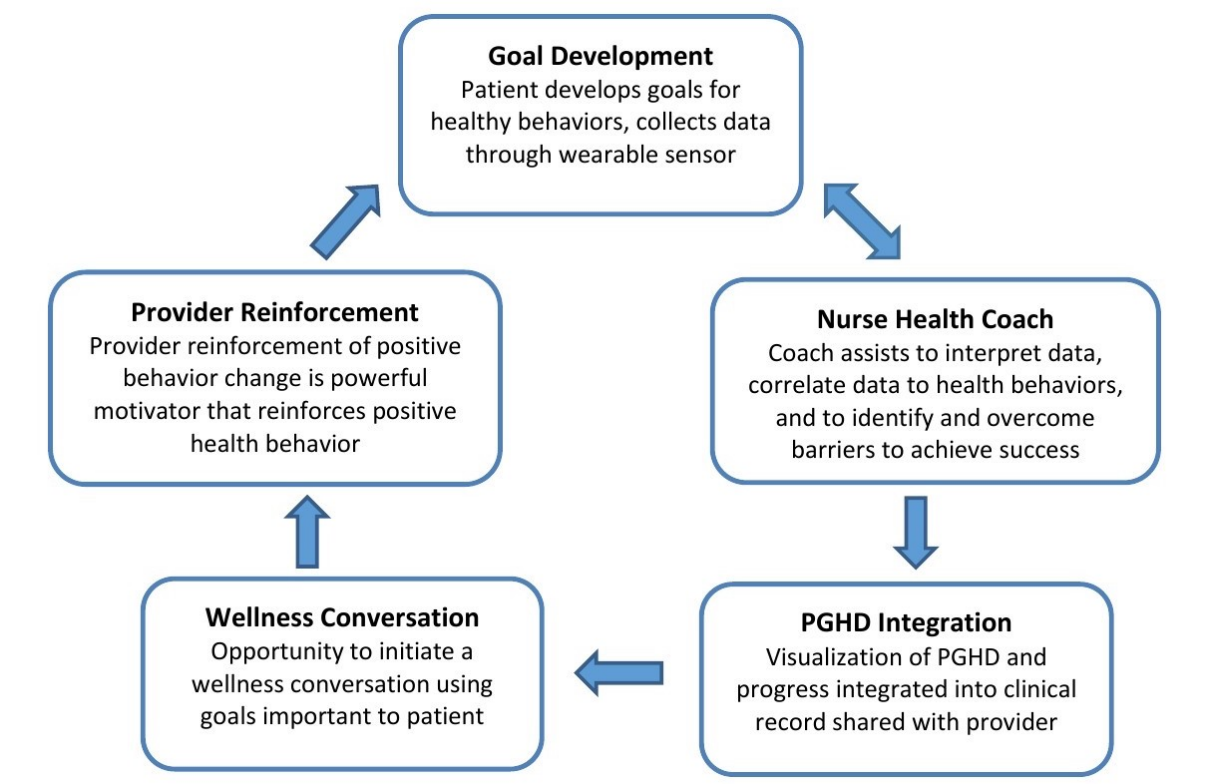
Changing the conversation about health. PGHD: patient-generated health data.

Prior to conducting an intervention study focused on improving exercise health and self-efficacy within an employee wellness program, our team conducted focus groups to understand potential users’ beliefs about the role of fitness trackers and nurse health coaching in supporting people to attain improved health behaviors. We conducted 4 focus groups with 30 employees of a large health system. Principal findings from this qualitative study elicited participant views that to create effective behavior change interventions, technology tools must go beyond tracking of PGHD. Participants identified the need for a health expert (nurse coach and/or primary care provider) to collaborate with them to create context and meaning from the data collected through an mHealth device. The following pathways to create meaning were identified: synthesizing data; helping to generate incremental, attainable goals; providing data-informed, tailored, and timely feedback; and provider investment in patient-centered behavior change work. A resulting model of how these design elements could ultimately change patient and provider engagement in health behavior change emerged from this work (Figure 1).

#### Engagement of Key Stakeholders in Intervention Design

This intervention program was developed with extensive input from patient, provider, and technology and informatics experts about key elements and considerations essential to build a program aimed at enhancing self-management success of persons living with diabetes. We invited the participation of three advisory boards: The Patient Advisory Board comprised of seven persons living with diabetes; the Provider Advisory Board comprised of 13 health care providers (primary care physicians, specialists, diabetes educators, and leaders from other health systems in the region); and the Technology Advisory Board comprised of 15 technology and informatics experts. Stakeholders met regularly with the research team. We brought data, prototype iterative designs, and results to our advisors to confirm relevancy of the findings, and to understand how to build an interface allowing for meaningful, right-sized, bidirectional data elements that complement and enhance current health system workflows.

### Essential Intervention Components

The P^2^E^2^T^2^ intervention design was finalized based on pilot study findings and input from key stakeholders. It is important to note that while all stakeholders discussed the value of blood glucose as a PGHD element, the decision was made to not include actionable data (requiring timely monitoring and provider intervention) in this initial demonstration to bring PGHD into the EHR. The core components of the intervention are: 1) the provision of a commercial sensor fitness tracking watch; 2) access to an existing mobile nutrition application; 3) regular nurse health coaching sessions over a period of three months; and 4) integration of PGHD and nurse coaching summaries into the EHR for provider, patient, and nurse coach reflection and tracking of progress.

## Methods

### Design

The P^2^E^2^T^2^ Program to Improve Health in Diabetes study is a prospective, randomized controlled trial conducted at an academic health system in California with 2 arms: 1) usual care offered through existing chronic disease management resources at the health system, and 2) the P^2^E^2^T^2^ Program. We planned to enroll 300 patients with type-2 diabetes from the health system’s primary care network, randomizing half to usual care and half to the P^2^E^2^T^2^ Program’s mHealth enabled nurse health coaching intervention. The study was approved by the University’s Institutional Review Board and was registered at ClinicalTrials.gov (NCT 02672176).

### Recruitment

**Figure 2:**
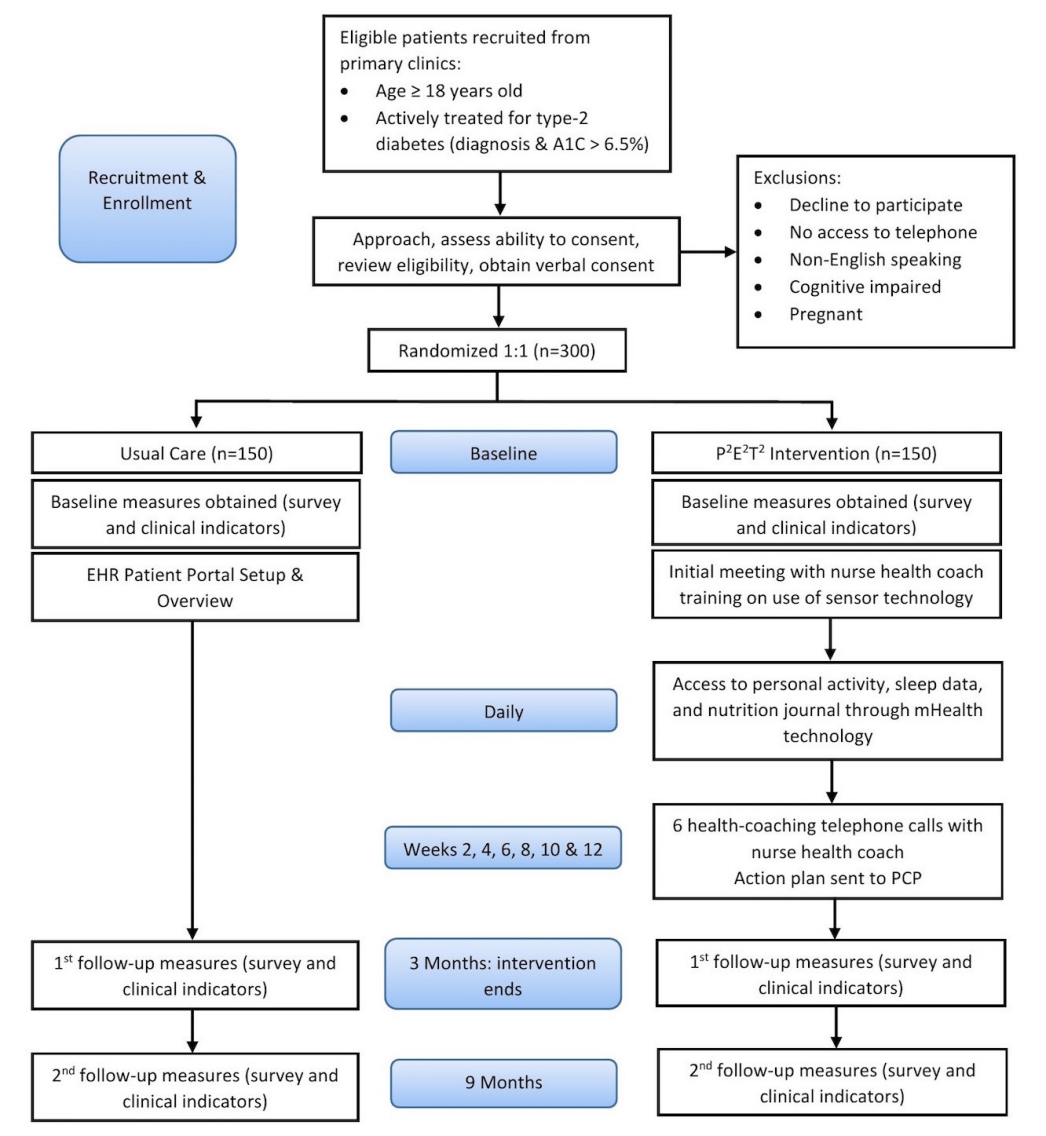
Diagram of Patient and Provider Engagement and Empowerment through Technology (P2E2T2) patient enrollment, randomization, intervention, and timeline. EHR: electronic health record; PCP: primary care provider.

Participants were recruited from three academic primary care clinics, 2 suburban and 1 hospital-based primary care clinic. Settings were purposely selected in an effort to enroll a diverse group of individuals living with diabetes. Eligibility criteria included: aged 18 years or older, living with diabetes mellitus (defined as diagnosis of diabetes mellitus type-2 and most recent HbA_1c_ lab test result of 6.5% or higher), enrollment at one of the participating primary care clinics, and able to speak English. Individuals were excluded if they did not have access to a telephone, did not speak English, were pregnant, or could not consent due to cognitive impairment (see Figure 2).

A query of the EHR using criteria for age, diagnosis, and clinic site generated a list of potentially eligible patients. Study information packets were mailed to individuals, including a brochure describing the study and an opt-out card. In the mailing, individuals were informed that a research team member would contact them by phone if they did not return the opt-out card to the study office within two weeks. To maintain confidentiality, the opt-out card identified participants only by an anonymous study identification number, did not include any personal information, and did not mention diabetes to ensure privacy of personal health information. Research staff made telephone calls to those who did not return an opt-out card 3 weeks after the mailing. With successful contact, a standardized script was used to describe the study, discuss expectations of participation, review eligibility, and answer any questions. Individuals who were interested in participating and met eligibility criteria were verbally consented and then randomized to a group.

### Group Allocation, Blinding, and Enrollment

Following verbal consent, block randomization to either usual care or intervention with a 1:1 ratio stratified by clinic site was accomplished using the Research Electronic Data Capture (REDCap), a secure Web research application designed to support data for research studies [16].

Participants were blind to their study group assignment as all participants received training in the use of health system technology offerings. Participants were assigned a unique personal identifier and a group identifier allowing for blinding at the point of analysis. Once randomized, participants were invited to attend an in-person group onboarding session according to their group allocation. At both control and intervention group sessions, participation expectations for the study were outlined, questions were answered, signed informed consent was obtained, and the baseline survey was completed on paper or tablet according to preference. All participants were informed of expectations for survey completion at baseline, 3 months, and 6 months.

### Control Group

During the onboarding session, participants received information and training on currently available technology (existing electronic patient portal) and diabetes related resources available at the health system. MyChart, a personal patient portal to the health system’s EHR system, was introduced and accounts were created for participants who did not already have an active account. Participants were shown how to access and use the MyChart portal through a smartphone, tablet, and/or personal computer. The features of MyChart, including making appointments, viewing labs, contacting providers, and accessing diabetes resources were reviewed. Usual care resources highlighted on the health system’s website and accessible to all patients living with diabetes at the health system (online educational tutorials, diabetes group classes, private messaging with a diabetes educator, and links to diabetes related websites), were reviewed with the participants. After the onboarding session, participants in the usual care group had no further contact with the study team other than reminders and prompting to complete survey measures at relevant intervals.

### Intervention Group

Participants randomized to the intervention arm received all elements of the orientation created for the control group, plus had an extended orientation to prepare them for the mHealth technology and nurse coaching components of the P^2^E^2^T^2^ intervention.

The P^2^E^2^T^2^ intervention consists of 3 components: 1) regularly scheduled telephone nurse health coaching sessions; 2) provision of a wireless sensor and mHealth application to capture physical activity, sleep, and nutrition data; and 3) integration of daily PGHD into the EHR.

#### Nurse Health Coaching

Participants in the intervention group were paired with a nurse health coach who collaborated with them to support health behavior changes. The goal of coaching is to promote mutual goal setting, track relevant health behavior data, and derive meaning from the data to reinforce and improve healthy choices (see Figure 1). An initial face-to-face meeting occurred during the onboarding session where participants met their assigned coach and learned about the coaching aspect of the intervention. Following the in-person meeting, telephone-coaching sessions were scheduled every 2 weeks for 3 months (6 contacts total) at times that were convenient for the participant. The initial coaching session elicited goals and motivations for improved health and established agreed upon metrics (eg, daily steps, calories, and carbohydrates) that the patient and nurse would track and discuss at subsequent telephone sessions. Nurses planned for 30-45 minutes for initial calls and 15-30 minutes for subsequent calls. With consent of participants, the coaching conversations were audio-recorded and uploaded to the study’s secure drive to monitor nurse health coach performance for quality assurance and intervention fidelity.

#### mHealth Technology

Each participant received a Garmin VivofitHR activity tracker watch. The watch captures real-time activity data, including steps taken, distance travelled, active calories burned each day, active minutes per week, heart rate, and hours of sleep at night. Participants can personalize goals and receive visual acknowledgement on the watch when they reach their goal for the day. MyFitnessPal, the nutrition tracking application, was an optional component installed on the iPhone or iPod to allow participants to log food and beverage consumption. Participants are able to view trends in activity level, sleep, and nutrition on their smartphone or computer. Participants were encouraged to wear and use the activity tracker for the entire 9-month duration of study participation. The study team made available technical support to participants by telephone throughout the intervention.

#### Patient-Generated Health Data Integration Into the Electronic Health Record

The data collected by the sensors synchronizes to either an iPhone or an iPod touch. Participants who did not have a compatible iPhone were given an iPod touch for use during the study. Apple HealthKit and MyChart were connectors that allowed PGHD to be automatically transmitted into the EHR for review by nurse coaches and the patient’s healthcare providers.

In order to facilitate the passive transfer of PGHD to the EHR, participants were encouraged to synchronize the activity tracker to their iPhone or iPod each day. Passive data transfer of PGHD between the mHealth technology and the EHR occurred for steps and calories burned. Calories and macronutrient information consumed by the participant and logged into the application were also transmitted into the EHR. At the time this study was initiated, it was not possible to passively transmit active minutes per week, sleep, weight, or nutrition data into the EHR, so participants were instructed to enter this data manually using MyChart if they wanted their coach or provider to have access to it.

EPIC is the EHR provider for the health system. We used an EPIC feature called Synopsis to design a single screen page to graphically display key PGHD elements in the EHR. Weight, activity, nutrition, and sleep PGHD can be individually selected and displayed in concert with clinically relevant data elements such as laboratory values, medications, and vital signs. Multiple authentication protocols were enacted by the patient and provider to authorize the collection and integration of sensor data into the EHR. The PGHD visualization dashboard within the EHR allowed the nurse and healthcare team the ability to view patient data collected by participants in their daily lives.

Summary documentation of coaching activities was also integrated in the EHR. After the final coaching call, the nurse coach sent a summary of each participant’s goals and achievements to his or her primary care provider. Participants were encouraged to continue goal setting and attainment to improve their health, to wear the fitness tracker, and to synchronize their PGHD with MyChart for an additional six months, coinciding with the study end date.

**Table 1:** Study outcome measure. Data for all variables collected at baseline, 3 months, and 9 months.

Variable	Source/Instrument
Self-efficacy	Survey, Diabetes Empowerment Scale – Short Form
Readiness to Change	Survey, Readiness to Change
HbA_1c_	Electronic health record abstraction
Quality of Life	Survey, Patient Health Questionnaire depression scale-9, Perceived Stress Scale, PROMIS (emotional distress, physical function and sleep disturbance)
Provider Satisfaction	Survey, Consultation and Relational Empathy Measure, Consumer Assessment of Healthcare Providers and Systems

### Sample size

Sample size goals were based on our previous randomized controlled trial of nurse coaching to improve disease self-management in which we found significantly higher self-efficacy scores in the nurse coaching intervention group compared to the control group [15]. A recruitment goal of 300 (150 for each arm) was established for this study based on both power and projected attrition. Attrition was 16% in the previous study using a similar intervention design with comparable demands on participants for time and response. Even under the conservative assumption that design effects and dropout rates could result in a reduced sample size of 100 per treatment group, the P^2^E^2^T^2^ study has at least 80% power to detect the specified clinically important effect size.

### Study Measures and Outcomes

The primary outcome of interest is self-efficacy, measured using the Diabetes Empowerment Scale–Short Form, a validated eight-item instrument designed to assess the psychosocial self-efficacy of people living with diabetes [17]. The Diabetes Empowerment Scale–Short Form includes items that address managing the psychosocial aspects of the diabetes, assessing dissatisfaction and readiness to change, and setting and achieving goals. Secondary outcomes of interest include changes in HbA_1c_, readiness to change, provider satisfaction [18,19], and quality of life measures [20-22]. Table 1 provides a complete list of variables collected and timing of collection.

### Data Collection

All measures were collected in both the intervention and usual care groups at three time points: baseline, three months (coinciding with intervention completion), and six months (selected to assess sustained effects of the intervention). At baseline, participants completed a demographic survey to assess age, gender, race, education level, income level, and health history. Participants received a $50 gift card for completion of each survey.

Surveys were emailed to study participants using REDCap. Paper surveys were available if preferred. Clinical data required to measure study outcomes were abstracted from the EHR and recorded in the REDCap study database by the nurse health coach at each data collection time point.

### Statistical analysis

Our analytic approach uses multivariate regression modeling for all hypothesis testing to estimate population trends and individual differences in change, such as those due to treatment effects. The mixed effects models include a main independent variable, a binary indicator for intervention assignment, and a parsimonious set of covariates to reduce the potential for confounding. Model fit will be based on deviance tests for nested models, the Akaike Information Criterion and the Bayesian Information Criterion for non-nested models. The estimates for the fixed effects will be assessed using a predetermined significance level (.05) on two-tailed tests and 95% confidence intervals. Intent-to-treat analysis will be used to assess the effect of the intervention by treating all eligible patients enrolled in the P^2^E^2^T^2^ program, regardless of intervention completion.

### Data Monitoring

All study personnel involved received Human Subjects Protection and Health Insurance Portability and Accountability Act compliance training. All data were managed and analyzed at the institution and maintained on secure servers accessible by individual login and password. In order to utilize commercially available mHealth devices it was necessary for the participants to share private and personal information with commercial technology vendors according to their privacy disclosures and terms of service.

## Results

The development phase of the study was completed and successful integration of PGHD into the EHR was in place prior to participant enrollment. The clinical trial was conducted between February 2016 and May 2017, with final data collected by December 2017 and final analyses and results anticipated by mid-2018.

## Discussion

This study combines evidence-based solutions for successful health improvement by offering nurse health coaching sessions paired with objective personal activity data collected through mHealth technology. We believe this is the first clinical trial to directly integrate and synchronize PGHD into the existing EHR of a large academic health system.

While type-2 diabetes is a progressive disease with a genetic component that in not modifiable, diabetes and associated comorbidities share common risk factors influenced by unhealthy behaviors. By focusing on health behavior goals identified by patients, the P^2^E^2^T^2^ program has the potential to improve both general health and quality of life. When individuals have the opportunity to take control of their health and make better behavioral decisions, they can directly prevent or mitigate the impact of chronic conditions. We hypothesize that greater personal and health outcomes can be achieved when the focus is on health goals prioritized and generated by the individual. The P^2^E^2^T^2^ program builds on traditional approaches to chronic disease management by providing tools and supports for individuals to accomplish patient-determined goals and bringing objective PGHD to the EHR to provide a more complete picture of efforts to improve and manage health. These data complement laboratory values and other clinical indicators and give a more complete and time-related summary of behavioral changes, such as physical activity, nutrition, and sleep. Together with clinical data, they offer an opportunity for a more comprehensive discussion about wellness.

There are several challenges in this approach. While there is growing consumer interest in personal technology devices and desire to integrate PGHD into chronic disease management, adoption is challenging for both patients and systems. This intervention depends on willingness and capacity to adopt technology as well as access to the technological tools. Technology use is on the rise across all age and socioeconomic groups, yet the digital divide persists, especially pertaining to readiness [23]. To address these challenges, we provided the necessary technology to individuals and developed extensive technology training and support materials. These materials were tested in advance with our patient advisory group for feedback and design improvement prior to dissemination. Lastly, the telephone support line staffed by research assistants to troubleshoot any technology or use issues by participants enhanced adoption of the technology.

The integration and use of PGHD into the secure and closed system of the EHR was a significant challenge. We believe this integration to be essential since technology that does not integrate with health systems' EHR was unlikely to be used by clinicians and providers. Given clinical time constraints, easy access and alignment with workflow is essential. Even though we have successfully integrated PGHD into the institution’s EHR, it will be important to identify barriers to integration into practice workflow during primary care visits.

### Conclusion

The P^2^E^2^T^2^ Program to Improve Health in Diabetes will serve as a resource to understand if the combination of objective mHealth gathered PGHD and nurse health coaching helps individuals with type-2 diabetes improve self-efficacy to manage their health. Integrating data generated by patients in their daily lives into the EHR allows for meaningful analysis of behavior choices and encourages patient-centered models of care to support and motivate patients to reach personal goals. It is imperative to the long-range plan of building systems and programs that, if found to be beneficial, this intervention can be designed to scale and translated to achieve outcomes in a larger population with various health challenges.

## Abbreviations

EHR: electronic health record
HbA_1c_: hemoglobin A_1c_
mHEALTH: mobile Health
P^2^E^2^T^2^: Patient and Provider Engagement and Empowerment through Technology
PCP: primary care provider
PGHD: patient-generated health data
REDCap: Research Electronic Data Capture
